# Supplementary material for: Comprehensive aortic stenosis characterization using multi-view deep learning
Source: medRxiv. 2025 Sep 29:2025.09.26.25336778. Preprint. [Version 1] doi: 10.1101/2025.09.26.25336778 (PMC12622131; doi:10.1101/2025.09.26.25336778)
Supplement: Supplement 1 [file NIHPP2025.09.26.25336778v1-supplement-1.pdf]

medRxiv preprint doi: https://doi.org/10.1101/2025.09.26.25336778; this version posted September 29, 2025. The copyright holder for this preprint (which was not certified by peer review) is the author/funder, who has granted medRxiv a license to display the preprint in perpetuity.

It is made available under a CC-BY 4.0 International license .

Supplementary Tables

sTable 1. Model performance to detect severe or at least moderate aortic stenosis.

Cohort or class	AUC	PPV	NPV	Recall	Specificity	F1
**Internal Test**						
≥ Moderate AS	0.955 (0.946 – 0.964)	0.842 (0.811 – 0.872)	0.909 (0.892 – 0.926)	0.828 (0.796 – 0.859)	0.917 (0.900 – 0.933)	0.835 (0.811 – 0.858)
Severe AS	0.964 (0.952 – 0.973)	0.763 (0.701 – 0.821)	0.960 (0.949 – 0.970)	0.729 (0.668 – 0.789)	0.966 (0.956 – 0.975)	0.746 (0.696 – 0.791)
**Temporal Test**						
≥ Moderate AS	0.983 (0.980 – 0.985)	0.823 (0.803 – 0.842)	0.979 (0.977 – 0.981)	0.762 (0.741 – 0.783)	0.985 (0.984 – 0.987)	0.791 (0.775 – 0.807)
Severe AS	0.985 (0.981 – 0.988)	0.731 (0.691 – 0.771)	0.989 (0.988 – 0.991)	0.636 (0.596 – 0.676)	0.993 (0.992 – 0.994)	0.680 (0.647 – 0.712)
**SHC**						
≥ Moderate AS	0.989 (0.984 – 0.993)	0.793 (0.740 – 0.843)	0.991 (0.987 – 0.995)	0.911 (0.872 – 0.948)	0.977 (0.970 – 0.983)	0.848 (0.811 – 0.882)
Severe AS	0.985 (0.975 – 0.992)	0.809 (0.723 – 0.885)	0.985 (0.980 – 0.990)	0.691 (0.600 – 0.777)	0.992 (0.988 – 0.996)	0.745 (0.673 – 0.809)
**CSMC**						
≥ Moderate AS	0.978 (0.974 – 0.982)	0.742 (0.702 – 0.781)	0.980 (0.977 – 0.983)	0.665 (0.624 – 0.706)	0.986 (0.983 – 0.988)	0.701 (0.667 – 0.733)
Severe AS	0.989 (0.986 – 0.992)	0.848 (0.797 – 0.895)	0.988 (0.986 – 0.990)	0.622 (0.565 – 0.678)	0.996 (0.995 – 0.998)	0.718 (0.670 – 0.763)

Performance metrics with 95% confidence intervals for the prediction of “severe AS” and “moderate or severe AS” across four cohorts. AUC, area under the receiver operating characteristic curve; PPV, positive predictive value, NPV, negative predictive value; ≥ Moderate AS, moderate or severe AS

medRxiv preprint doi: https://doi.org/10.1101/2025.09.26.25336778; this version posted September 29, 2025. The copyright holder for this preprint (which was not certified by peer review) is the author/funder, who has granted medRxiv a license to display the preprint in perpetuity.

It is made available under a CC-BY 4.0 International license .

sTable 2. Severe AS detection performance in CSMC replicated cohort.

Models	
	CSMC replicated cohort (N=4226)
**EchoNet-AS**	0.972<br>(0.963 – 0.979)
Ensemble of B Modes + Color Dopplers	0.971<br>(0.961 – 0.978)
Ensemble of B Modes	0.968<br>(0.958 – 0.976)
PLAX	0.956<br>(0.940 – 0.969)
PLAX (Yale model, value from published paper[11])	0.952<br>(0.941 – 0.963)
